# Lost in Transition: A Systematic Review of Neonatal Electroencephalography in the Delivery Room—Are We Forgetting an Important Biomarker for Newborn Brain Health?

**DOI:** 10.3389/fped.2017.00173

**Published:** 2017-08-10

**Authors:** Daragh Finn, Eugene M. Dempsey, Geraldine B. Boylan

**Affiliations:** ^1^Department of Paediatrics and Child Health, University College Cork, Cork, Ireland; ^2^Irish Centre for Fetal and Neonatal Translational Research, University College Cork, Cork, Ireland

**Keywords:** newborn, electroencephalography, neuro-monitoring, delivery room, hypoxic–ischemic encephalopathy, prematurity

## Abstract

**Background:**

Electroencephalography (EEG) monitoring is routine in neonatal intensive care units (NICUs) for detection of seizures, neurological monitoring of infants following perinatal asphyxia, and increasingly, following preterm delivery. EEG monitoring is not routinely commenced in the delivery room (DR).

**Objectives:**

To determine the feasibility of recording neonatal EEG in the DR, and to assess its usefulness as a marker of neurological well-being during immediate newborn transition.

**Methods:**

We performed a systematic stepwise search of PubMed using the following terms: infant, newborns, neonate, DR, afterbirth, transition, and EEG. Only human studies describing EEG monitoring in the first 15 min following delivery were included. Infants of all gestational ages were included.

**Results:**

Two original studies were identified that described EEG monitoring of newborn infants within the DR. Both prospective observational studies used amplitude-integrated EEG (aEEG) monitoring and found it feasible in infants >34 weeks’ gestation; however, technical challenges made it difficult to obtain continuous reliable data. Different EEG patterns were identified in uncompromised newborns and those requiring resuscitation.

**Conclusion:**

EEG monitoring is possible in the DR and may provide an objective baseline measure of neurological function. Further feasibility studies are required to overcome technical challenges in the DR, but these challenges are not insurmountable with modern technology.

## Introduction

Electroencephalography (EEG) has become a routine component of neurological monitoring in the neonatal intensive care unit (NICU) (1, 2). It has well-documented benefits in monitoring newborn infants with perinatal asphyxia (3–6) and seizures (7–13) and in predicting long-term outcome (14–21). Five to ten percent of newborn infants require some measure of stabilization in the delivery room (DR) (22). The majority of infants who require complex stabilization are either extremely premature or have sustained birth asphyxia. In recent decades, the survival rates for both preterm and asphyxiated infants have improved, but neurodevelopmental morbidity has not decreased in corresponding order (23, 24). An increased focus on the early identification and prevention of brain injury in newborn infants is now a major focus of newborn care in the setting of the NICU. However, neurological monitoring is not routine during newborn stabilizations in the DR, nor is it recommended in recent international guidelines (25, 26). First, we will discuss the current methods for assessing brain health in the DR, and then we outline our rationale for considering EEG as a very useful biomarker of brain health in the DR. Table 1 summaries the different assessment tools discussed.

**Table 1:** Current and possible future tools for assessing neonatal brain health in the delivery room (DR).

	Method	Strengths	Limitations
Clinical assessment	Muscle tone and reflex irritability as part of the APGAR score	Immediate score	Subject to inter- and intra-rater variability

Cerebral blood flow	Ultrasound Doppler of cerebral or carotid artery	Immediate assessment of cerebral blood flow	Technically challenging and continuous data acquisition not feasible

Near infrared spectroscopy (NIRS)	Non-invasive monitoring of cerebral tissue oxygenation by application of NIRS pad to frontal area	Feasible to obtain continuous reliable data in the DR	Wide range for normative values
		Normative values established	

Fetal electroencephalography (EEG)	Application of >1 EEG electrodes to fetal scalp during labor	Would allow for real time assessment of fetal brain health	Technically challenging
			Can only be applied during late stages of labor
			Not established as method for assessing fetal health
			Paucity of normative data

EEG	Application of >1 EEG electrodes to neonatal scalp after delivery	Would allow for real time assessment of neonatal brain health	Technically challenging
			Paucity of normative data
		Established method for monitoring neonatal brain health in neonatal care	

## Current Methods for Assessing Brain Health in the DR

At present, neonatal stabilization teams rely on clinical parameters to assess a newborn infant’s neurological status during immediate newborn transition. Assessments of muscle tone and reflex irritability are incorporated into the Apgar score, which is routinely assigned to infants after 1 and 5 min (27, 28). However, Apgar scores are subjective and inter-rater variability is high (29). Neonatal objective hemodynamic monitoring with reliable, continuous, non-invasive measurements of physiological parameters such as heart rate and pre-ductal oxygen saturations (with pulse oximetry) is now routine in the DR (29–32). However, neonatal stabilization teams do not have objective information available about neurological function during resuscitation. The availability of accurate and objective baseline neurological information may help guide resuscitation and plan appropriate early interventions for neonates that may not have tolerated the stresses of labor so well.

Studies that have previously sought to introduce neurological monitoring into the DR initially focused on cerebral blood flow using Doppler measurements of cerebral or carotid arteries (33–38). More recently, studies have concentrated on near infrared spectroscopy (NIRS), which provides non-invasive monitoring of cerebral tissue oxygenation in the DR (39–46). Guidelines for the use of NIRS monitoring and EEG in NICUs overlap, and it is advised that they should be used simultaneously (6).

## Rationale for Proposing EEG as a Biomarker of Newborn Brain Health in the DR

Electroencephalography is not a new technique, but its application in neonatology in the past has been hampered by a lack of appropriate technology for recording and analysis. This has changed dramatically in the last decade, and there are now high quality digital amplifiers available that can record excellent EEG signals even in very noisy environments. The time is now right to reexplore the use of EEG as a valuable biomarker of neurological function in the DR; an environment where previously, it was just not possible.

The signal measured by the EEG is of the order of microvolts and represents a direct measure of postsynaptic neuronal activity in the cortex. Research has shown that the EEG of fetal sheep can be recorded during labor (47–49). Thaler and colleagues performed intrapartum EEG on fourteen women with uncomplicated pregnancies (50), and a clinical trial of EEG monitoring during labor is currently underway (https://clinicaltrials.gov/ct2/show/NCT03013569). During normal labor, the fetus is exposed to brief but repeated episodes of hypoxia, which are balanced by the fetus’s striking ability to adapt to these episodes (51). Fetal EEG monitoring in both human and animal studies during labor has shown that these episodes are associated with rapid EEG amplitude reduction and also with fast amplitude recovery as soon as the uterine contraction ends (48, 52). The EEG is exquisitely sensitive to any impairment in oxygen delivery to the brain. A reduction in oxygen leads to an immediate suppression of synaptic transmission with a reduction (often complete suppression) in EEG amplitude (48, 53). This adaptive response, believed to be mediated by multiple inhibitory neuromodulators including adenosine, to hypoxia may be protective by decreasing energy consumed by the generation of synaptic potentials (54). If cerebral hypoxia is sustained, however, EEG amplitudes remain severely reduced and membrane failure will eventually occur accompanied by energy depletion and cell damage (52). Thus, sustained changes in the EEG signal a risk of impending brain injury.

In neonates with hypoxic–ischemic encephalopathy (HIE), an EEG showing sustained suppression for hours after birth has long been associated with a very poor outcome (5, 15, 55, 56). Neonatal EEG monitoring is recommended for all infants with moderate and severe HIE, and neonatal teams are now familiar with its application in NICUs. Fetal EEG monitoring has clear benefits for the early recognition of HI injury but requires considerable research before it is adopted as a routine tool for fetal surveillance. Immediate EEG acquisition in the DR on the other hand is much more feasible and may quickly identify those neonates that have not tolerated labor and delivery very well, which will be seen as disrupted patterning on the EEG.

An early EEG in the DR of an infant requiring resuscitation will indicate if EEG activity is present or not or if EEG activity returns following this stabilization process. As we know that EEG activity should recover immediately following restoration of oxygen delivery to the brain, if EEG activity does not return immediately post resuscitation or activity is severely disrupted, this indicates that the infant is at risk of hypoxic–ischemic brain injury. This could provide a clear indication for immediate passive cooling prior to transfer to the NICU. This early indication of cerebral function is very important as Thoresen et al. have shown that infants cooled within 3 h of birth have better neurodevelopmental outcomes when compared to infants whose cooling commenced between 3 and 6 h (57). Further improvements in outcome are highly likely to arise from earlier improved identification of affected infants that would allow earlier initiation of treatment after resuscitation.

Therefore, early EEG monitoring could provide neonatal stabilization teams with valuable, much needed, information about the neurological status of the newborn infant immediately after birth. Thus, we set out to assess whether any studies had already attempted to measure the human EEG in the DR by conducting a systematic review of available literature. We also aimed to establish the feasibility of EEG monitoring in the DR, and determine whether valuable information has been acquired from its application thus far.

## Methods

### Search Strategy

We performed a systematic stepwise search of PubMed as per the Preferred Reporting Items for Systematic Reviews and Meta-Analyses (58). Articles up to and including February 2017 were included. Studies had to involve EEG monitoring in the DR. Search terms included the following: infant, newborns, neonate, DR, afterbirth, transition, and EEG. Only human studies were included, and this was incorporated into the initial search. Additional published reports identified in review articles or referenced in articles screened were also included. Publication bias was not assessed.

### Study Selection

Articles identified by our search strategy were screened for inclusion by one author (DF). Titles and abstracts were initially screened. Articles had to pertain to EEG monitoring immediately after birth. Studies that focused on infants post birth asphyxiation or infants who had intracranial pathology were excluded as the subjects were, by nature, recruited post-delivery and not relevant to our search. Studies that specified a time frame for initial EEG monitoring outside of the first 15 min of life, or initial recruitment outside of the DR were also excluded. Where uncertainty remained regarding eligibility for inclusion the full text was reviewed. Studies that were not available in English were excluded.

## Results

**Figure 1:**
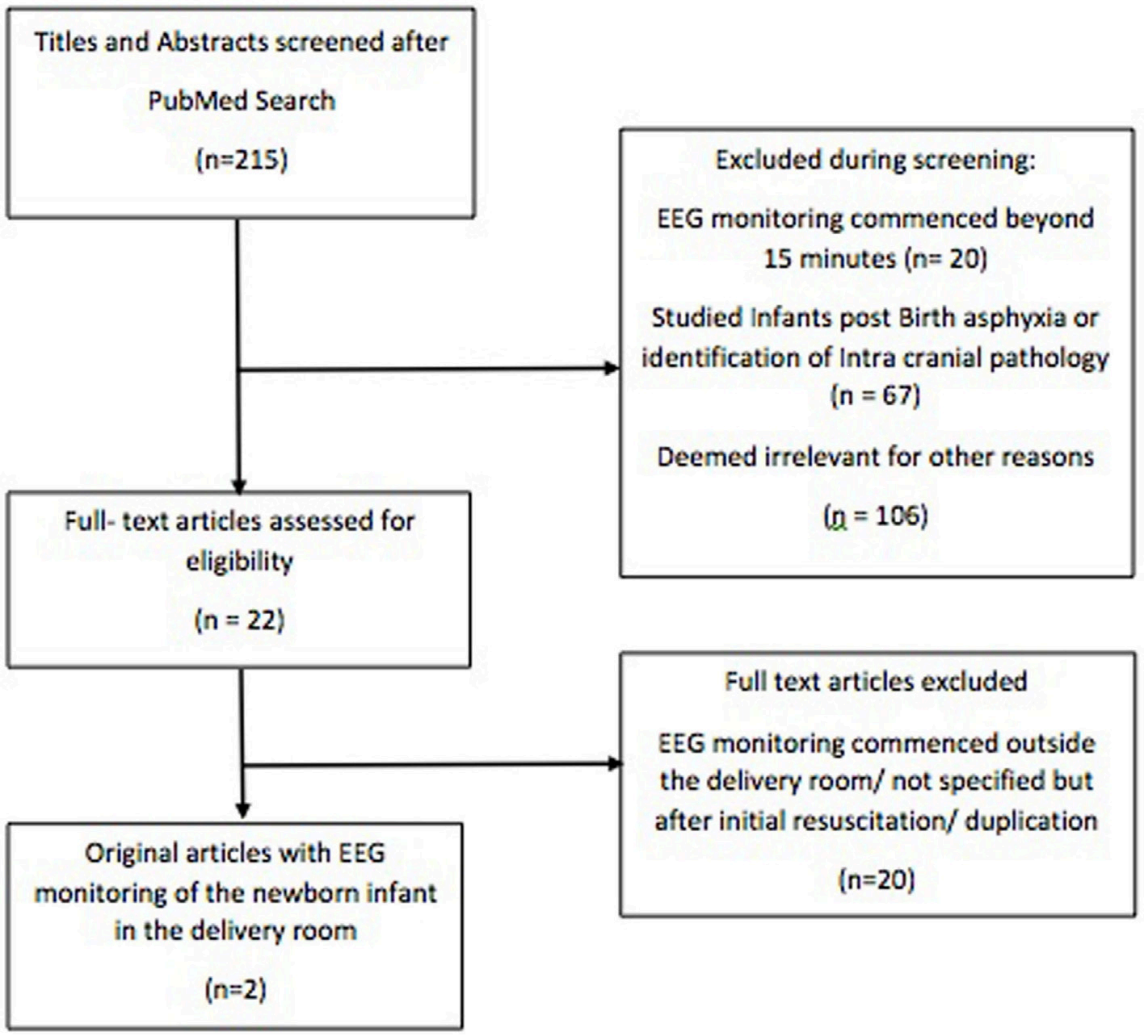
Flow diagram of literature search.

Our initial search identified 215 articles (see Appendix 1 in Supplementary Material). After assessment of these articles, two original studies were identified that described EEG monitoring of the newborn infant within the DR (Figure 1). One study also contributed to a review article identified by our search, which was excluded from our study to avoid duplication (59). Table 2 summarizes the two studies identified.

**Table 2:** Summary of electroencephalography (EEG) studies in the delivery room.

Reference	Neonates	Number recruited and monitored	Design	Number included in analysis	Observation
Pichler et al. (42, 60)	>34 weeks	46	Observational Amplitude-integrated EEG (aEEG) analysed for minimum and maximum voltages Near infrared spectroscopy (NIRS)	*N* = 46 31 uncompromised 15 required respiratory support	No significant differences between minimum and maximum voltages when the 2 groups are compared Uncompromised infants had higher V max in minute 3 and 4 compared with minute 10

Tamussino et al. (61)	Term	244	Observational aEEG analysed for minimum and maximum voltages Infants with initial low voltages which normalized were compared to infants with normal voltages throughout NIRS	*N* = 59 9 met inclusion criteria 50 control studies	Neonates with initially low cerebral activity during immediate transition after birth displayed lower cerebral saturations (<10th percentile) on NIRS, but increased cerebral oxygen extraction (cFTOE >90th percentile)

Pichler et al. performed a prospective observational study of infants born by elective cesarean section over 34 weeks gestational age (60). Infants at lower gestational ages were excluded due to concerns about their small head size, and the feasibility in applying EEG leads and NIRS to a small surface area. Four gold electrodes (two frontal and two parietal) were applied with contact gel, along with an NIRS pad to the left forehead, and overlying elastic bandages for support. Amplitude-integrated EEG (aEEG), a rectified, filtered, and compressed form of EEG, was acquired and stored. Overall, they found that aEEG monitoring of the newborn infant in the DR is feasible, but it is difficult to obtain continuous reliable data. Of a total number of 63 infants, 17 (27%) were excluded due to unreliable data. Of the remaining 46 infants, no data were recorded prior to 3 min of delivery, 25% had data available at 3 min, and just over 50% were available at 5 min. aEEG data were analyzed for mean minimum and mean maximum voltages every minute, and then correlated with cerebral oxygenation, heart rate and pre-ductal oxygen saturations. Findings were then compared between infants who were uncompromised at birth (*n* = 31) and infants who required neonatal resuscitation (*n* = 16).

Different cerebral activity patterns were identified between uncompromised newborns and those requiring resuscitation. They reported that infants in the uncompromised transition group started with initially high voltages on aEEG, followed by a significant decrease to baseline voltages at 4–5 min. In contrast, infants in the group requiring respiratory support did not show this pattern. However, there were no significant differences between minimum and maximum voltages when the two groups were compared, which the authors attribute to low numbers in the respiratory support group.

Tamussino et al. recorded simultaneous aEEG and NIRS in 244 term neonates during the first 15 min after delivery (61). Similar to the study of Pichler et al., aEEG data were analyzed for mean minimum and mean maximum voltages every minute, and then correlated with cerebral oxygenation, heart rate, and pre-ductal oxygen saturations. Neonates with initial low voltages, which normalized during transition, were compared to neonates with normal aEEG values throughout the monitoring period. Nine neonates had low initial aEEG voltages and were compared to 50 neonates with normal aEEG voltages throughout. Therefore, of 244 infants recruited, 59 aEEG recordings were included in the analysis. Neonates with initially low cerebral activity during immediate transition after birth displayed lower cerebral saturations (<10th percentile) on NIRS, but increased cerebral oxygen extraction (cFTOE >90th percentile). The authors concluded that neuro-monitoring with aEEG and NIRS might provide useful information on the neonates’ condition during immediate transition.

## Discussion

Neonatal mortality has decreased significantly in recent decades (24). As more infants survive following preterm delivery and birth asphyxia, achieving the best possible neurological outcomes for survivors is paramount. Whilst EEG has an essential role within the NICU in newborn neurological monitoring following birth asphyxia, and in monitoring preterm infants, it is not routinely initiated in the DR, and at present has no role during newborn stabilizations. We set out to determine if first it was feasible to perform newborn EEG in the DR, second to assess what information it provides about newborn brain activity in the immediate postnatal period and most importantly, to determine if this early objective information about brain activity would be useful. We found two studies that had performed aEEG during the first 15 min of birth. They found aEEG to be feasible in infants >34 weeks, but technically difficult to obtain continuous reliable data. Patterns of brain activity differed between infants that required newborn stabilization measures and infants that transitioned from the fetal to neonatal period without the need for medical intervention (60). Also, newborn infants with initially low cerebral activity during immediate transition after birth displayed lower cerebral saturations and increased cerebral oxygen extraction, compared with normal voltages throughout (61). The authors proposed a number of possible explanations for the differences between groups. They postulated that apnea, respiratory distress, and bradycardia in the immediate newborn may result in a lower cardiac output and resultant lower brain activity in compromised infants (60). For uncompromised infants who had initially high levels of activity, they suggested that catecholamine release may be responsible (62).

The brain is the most vulnerable organ in newborn infants. A non-invasive, continuous method to measure cerebral activity (EEG) is already available but it has not transitioned to the DR. Initial research focused on cerebral blood flow measurements but they were found to be technically difficult and did not provide continuous data (59). NIRS has shown great promise in providing continuous data on cerebral tissue oxygenation values. It utilizes the transparency of biological tissue to light in the near infrared spectrum to measure cerebral tissue oxygenation (63). A number of studies have examined cerebral oxygenation using NIRS in the DR (59), and recently, normative values for infants not requiring resuscitation have been published (42).

As the importance of the early instigation of neuroprotective strategies for term newborns with perinatal asphyxia has become evident, EEG monitoring (usually aEEG) has become more common in NICUs (1, 7). In contrast to cerebral blood flow and NIRS, EEG has well documented applications in the clinical management of newborn infants. It is the gold standard method for the accurate detection of all neonatal seizures in term and preterm infants (6, 9). It has well-proven efficacy in predicting outcomes following perinatal asphyxia, based on patterns of poor background activity and the timing of sleep wake cycling reestablishment (14, 15, 64, 65). Prediction of outcome following preterm delivery is more complicated, but investigations are ongoing (20). Several studies have shown that early background EEG suppression correlates with severity of periventricular hemorrhage (66–68). Also, continuous displays of inter-burst interval duration, which differs with gestational age, may become a useful prognostic measure in preterm infants in the near future (69, 70).

Despite its importance in monitoring the newborn brain in the NICU, EEG monitoring in the DR is currently not recommended. Stabilization of newborn infants in the DR, including infants with perinatal asphyxia, occurs without any objective measure of brain activity, and we found only two studies that have assessed the feasibility of obtaining a newborn EEG recording in the DR. Both studies used the aEEG trend and both found it possible to obtain aEEG tracings within 3 min in some cases, but obtaining continuous reliable data was generally difficult (60). Within these limitations the authors describe different patterns in brain activity for infants that required respiratory support and infants that transitioned independently. Also, aEEG was correlated with different cerebral oxygenation patterns. These findings are important as they pave the way for future studies.

Both studies analyzed brain activity by interrogating the aEEG mean minimum and mean maximum voltages. However, the aEEG trend alone is a high level summary measure of the EEG with poor time resolution due to compression in the aEEG algorithm, and it does not display the second by second activity of the brain; as a result, it is not optimal for application in the DR. Digital aEEG machines obtain one or two channels of EEG signal, which is then amplified and passed through an asymmetric band-pass filter that strongly attenuates activity less than 2 Hz and more than 15 Hz, to minimize artifacts. Additional processing includes semilogarithmic amplitude compression, rectification, and time compression (13). Heavy signal processing used in the aEEG algorithm eliminates much of the detail (e.g. frequency band content) available in the EEG, and many clinically important features are lost. Furthermore, there is no clear definition for aEEG, and most EEG machines implement different versions of the aEEG algorithm (71). The mean and maximum of the aEEG voltage need to be plotted and displayed for a number of minutes before any assessment of the overall baseline EEG activity can be made. In addition, it is well known that interpretation of the background aEEG pattern can be problematic due to baseline drift and other artifacts (72, 73). This is not optimal for DR EEG recording when real-time second by second information would be advantageous. For example, a recording of approximately 30 s duration alone using standard EEG would be enough to establish the presence of continuous EEG activity in a term newborn. This information would be hugely beneficial in the DR to help guide resuscitation and to determine the need for immediate passive cooling. Thoresen et al. coined the phrase “time is brain” in relation to the timing of cooling for neuroprotection (57), and we strongly believe that EEG in the DR could help identify those infants who would benefit most from early neuroprotective strategies.

Whether EEG could play a role in prognostication for infants requiring TH in the immediate newborn period is less clear. From clinical and preclinical studies, we know that recovery of EEG activity during the first 24 h after hypoxia ischemia, after a period of prolonged (several hours) suppression, can be associated with normal outcome (74, 75), and little to no histological injury (76). However, we now know that infants with even mild HIE can have cognitive delays at 5 years (14). The prolonged suppression is an actively mediated response, at least partially mediated by neurosteroids such as pregnanes and adenosine, which are upregulated for hours after the insult (77, 78). Previous papers that reported normal outcomes in infants with an initial flat EEG trace that recovered quickly and had normal outcome were limited by small numbers and follow-up continued until 2 years of age (75). The authors even admit this themselves as they say that “a normal score in the early years cannot preclude later neurological, perceptual-motor, or cognitive abnormalities” (75). Therefore, we continue to recommend multichannel continuous EEG monitoring for such infants for the duration of TH.

Electroencephalography in its raw format (not a modified aEEG) can be assessed both qualitatively and quantitatively. Qualitative EEG analysis is mainly used for clinical purposes. It is based on visual interpretation of the EEG signal and describes background features such as amplitude, frequency, and continuity of the EEG, symmetry, synchrony, and sleep–wake cycling. Quantitative EEG analysis is a method predominantly used in research and includes time and frequency domain analysis. Neither study identified in our review analyzed the EEG in its raw format, either for qualitative or quantitative purposes.

However, we still have a way to go before EEG monitoring is routine in the DR. Signal interpretation is difficult, but huge advances have already been made in quantitative analysis of the neonatal EEG and we now have algorithms that can accurately grade the EEG in term and preterm newborns (70, 79–84). The feasibility of EEG recording is constantly improving and newer amplifiers with high common mode rejection ratios are now available that make EEG recording more possible and less susceptible to noise and other artifacts. We have seen that excellent quality EEGs are now possible for even extremely preterm infants in the NICU, as long as there is adequate setup and preparation (85). Multiple channels of EEG are not required to assess the grade of EEG baseline activity in the DR, one channel of good quality EEG is perfectly acceptable to assess amplitude, continuity, and frequency of the EEG. EEG sensors are constantly evolving, and newer disposable single application sensors are now available also making EEG electrode acquisition more feasible.

Electroencephalography has long been considered just too difficult to deploy in environments like the DR and NICU. There have been major recent advances to the adoption of EEG in the NICU primarily due to advances in technology (2). Modern machine learning techniques are also advancing rapidly and will soon be able to provide non-EEG experts with the help needed to assist in the interpretation of EEG patterns on a 24/7/365 basis. These difficulties should no longer be a barrier to the adoption of EEG in the DR.

In conclusion, the time is now right to advance the objective monitoring of neurological function of newborn infants in the DR, and urgent research is clearly warranted. More EEG studies from healthy term and preterm newborns in the DR to establish feasibility and normative reference ranges are clearly a priority. Advances in automated analysis of the baseline EEG will be hugely beneficial in this effort particularly if outputs are incorporated into standard patient monitors. We look forward to further studies in this area.

## Author Contributions

GB and ED conceived and designed the review. DF performed the literature search and drafted the initial manuscript. All the authors (DF, ED, and GB) critically revised the manuscript for important intellectual content, agreed on the final manuscript, and approved its submission for publication.

## Conflict of Interest Statement

The authors declare that the research was conducted in the absence of any commercial or financial relationships that could be construed as a potential conflict of interest.
