# Supplementary material for: Lost in Transition: A Systematic Review of Neonatal Electroencephalography in the Delivery Room—Are We Forgetting an Important Biomarker for Newborn Brain Health?
Source: Front Pediatr. 2017 Aug 10;5:173. doi: 10.3389/fped.2017.00173 (PMC5554119; doi:10.3389/fped.2017.00173)
Supplement: Supplementary file 1 [file data_sheet_1.docx]

**Appendix 1**

Search Strategy for PubMed:

No limits activated

#1 MeSH descriptor Infant, Newborns explode all trees (Result #506,508)

#2 MeSH descriptor Neonate explode all trees (Result #506,508)

#3 MeSH descriptor Delivery Room explode all trees (Result #1,235)

#4 Title/Abstract Delivery Room (Result #1,542)

#5 MeSH descriptor Delivery Suite (Result #1,235)

#6 Text word After birth (Result # 32,243)

#7 Text word Transition (Result #222,046)

#8 MeSH descriptor Electroencephalography (Result #127,481)

#9 #8 with limits added for Human only studies (Result #95,980)

#10 #1 OR #2 (Result #491,669)

#11 #3 OR #4 OR #5 OR #6 OR #7 (Result #87,960)

#12 (#10) AND (#11) (Result #13,751)

#13 (#9) AND (#12) (Result # 215)

(("Infant, Newborn"[Mesh] OR "infant, newborn"[MeSH Terms]) AND (((transition[Text Word] OR after birth[Text Word]) OR "delivery rooms"[MeSH Terms]) OR delivery room[Title/Abstract])) AND ("electroencephalography"[MeSH Terms] AND "humans"[MeSH Terms]) AND "humans"[MeSH Terms]
